# DNA-testing for *BRCA1/2* prior to genetic counselling in patients with breast cancer: design of an intervention study, DNA-direct

**DOI:** 10.1186/1472-6874-12-12

**Published:** 2012-05-08

**Authors:** Aisha S Sie, Liesbeth Spruijt, Wendy AG van Zelst-Stams, Arjen R Mensenkamp, Marjolijn J Ligtenberg, Han G Brunner, Judith B Prins, Nicoline Hoogerbrugge

**Affiliations:** 1 Department of Human Genetics, Radboud University Nijmegen Medical Centre, Nijmegen, the Netherlands; 2 Department of Medical Psychology, Radboud University Nijmegen Medical Centre, Nijmegen, the Netherlands

**Keywords:** Hereditary, Breast cancer, *BRCA*, Genetic, Counseling, DNA

## Abstract

**Background:**

Current practice for patients with breast cancer referred for genetic counseling, includes face-to-face consultations with a genetic counselor prior to and following DNA-testing. This is based on guidelines regarding Huntington’s disease in anticipation of high psychosocial impact of DNA-testing for mutations in *BRCA1/2* genes. The initial consultation covers generic information regarding hereditary breast cancer and the (im)possibilities of DNA-testing, prior to such testing. Patients with breast cancer may see this information as irrelevant or unnecessary because individual genetic advice depends on DNA-test results. Also, verbal information is not always remembered well by patients. A different format for this information prior to DNA-testing is possible: replacing initial face-to-face genetic counseling (DNA-intake procedure) by telephone, written and digital information sent to patients’ homes (DNA-direct procedure).

**Methods/design:**

In this intervention study, 150 patients with breast cancer referred to the department of Clinical Genetics of the Radboud University Nijmegen Medical Centre are given the choice between two procedures, DNA-direct (intervention group) or DNA-intake (usual care, control group). During a triage telephone call, patients are excluded if they have problems with Dutch text, family communication, or of psychological or psychiatric nature. Primary outcome measures are satisfaction and psychological distress. Secondary outcome measures are determinants for the participant’s choice of procedure, waiting and processing times, and family characteristics. Data are collected by self-report questionnaires at baseline and following completion of genetic counseling. A minority of participants will receive an invitation for a 30 min semi-structured telephone interview, e.g. confirmed carriers of a *BRCA1/2* mutation, and those who report problems with the procedure.

**Discussion:**

This study compares current practice of an intake consultation (DNA-intake) to a home informational package of telephone, written and digital information (DNA-direct) prior to DNA-testing in patients with breast cancer. The aim is to determine whether DNA-direct is an acceptable procedure for *BRCA1/2* testing, in order to provide customized care to patients with breast cancer, cutting down on the period of uncertainty during this diagnostic process.

**Trial registration:**

The study is registered at the Dutch Trial Registry http://www.trialregister.nl (NTR3018).

## Background

Patients with breast cancer at high risk of an underlying hereditary predisposition face a time-consuming diagnostic process of several months: it might be helpful to be able to cut down on this long period of uncertainty and provide information applicable to their personal situation as early as possible. Having personal experience with breast cancer, these patients are likely to have enhanced personal risk estimates, thus a higher expectation for protective actions such as longer or more intensive surveillance [1]. Should they carry a pathogenic mutation in either the *BRCA1* or *BRCA2* gene, these patients do have a considerable long term risk for developing a second primary breast cancer (either ipsi- or contralateral) of up to 60% [2-4]. Women recently diagnosed with breast cancer may want to take their *BRCA1/2* status into consideration for their choice of surgical treatment (i.e. breast-conserving with radiotherapy versus ipsi/contralateral mastectomy) and, in the near future, chemotherapy (i.e. PARP-inhibitors) [5-10]. *BRCA1/2* mutation carriers face an additional risk of ovarian cancer ranging from 20-60% for *BRCA1* and 2-20% for *BRCA2*[2-4]. As screening for ovarian cancer through yearly serum CA-125 measurements and transvaginal ultrasound has proven to be ineffective [11-13], clinicians strongly recommend prophylactic bilateral salpingoophorectomy (pBSO) around the age of 35 to 40 years [13]. pBSO reduces the risk of ovarian cancer by 80-90%, and in unaffected premenopausal women simultaneously reduces breast cancer risk by 50% [14,15].

Patients with breast cancer often express concern and uncertainty regarding the risk of breast cancer for their unaffected relatives, especially their sisters and daughters [16]. For unaffected relatives carrying a *BRCA1/2* mutation, cumulative breast cancer risk at the age of 70 years ranges from 40-80% [2-4]. At the age of 25 years, they may choose between an intensive breast cancer screening program consisting of yearly MRI scans, mammography and clinical breast examinations [17,18] or undergoing prophylactic surgery, reducing the risk for breast cancer by 90% [19-21]. Some carriers may still be at an age to be confronted with childbearing conflicts [22]. These are only a few of the life-changing decisions for both patients with breast cancer and their relatives dependent on the results of DNA-testing, which may or may not confirm the presence of a genetic predisposition for breast and ovarian cancer. A previous study in Dutch patients being evaluated for possible breast cancer showed that these patients experienced the period before the final diagnosis as the most stressful, regardless of whether they had received a benign or cancer diagnosis afterwards [23]. This same principle likely applies to *BRCA1/2* testing. Reducing the period of uncertainty in the diagnostic process and offering various forms of information might help substantially.

Another attempt to speed up the diagnostic process concerning hereditary cancer was previously introduced in the evaluation of hereditary colon cancer in the Netherlands. Pathologists are now able to test tumor material of patients younger than 50 years for microsatellite instability (MSI) and immunohistochemical staining of gene products, which may reveal a high a priori risk for an underlying genetic predisposition, without prior consultation of a genetic counselor. If these characteristics are present, patients are referred for further evaluation by a genetic counselor [24,25]. This so-called MIPA procedure (MSI-test by pathologists) is seen by patients as a valuable addition to the diagnostic process of hereditary colon cancer, without feeling either overwhelmed or underinformed, nor showing increased levels of psychosocial distress [26-28].

Such an intervention may also be applicable to patients with breast cancer. As there is no equivalent of tumor material testing in hereditary breast cancer, alternatives for modification must be found within the current diagnostic process. Genetic testing for hereditary breast cancer includes genetic counseling both prior to and following DNA-testing, based on guidelines regarding presymptomatic testing for Huntington’s disease (HD) [29-31]. This approach had been adopted for hereditary breast cancer due to concerns about the psychological consequences of *BRCA1/2* genetic susceptibility testing [32]. However, extensive prior research shows there is no significant long term psychological impact: after an initial increase following *BRCA1/2* testing, psychosocial distress returns to pre-testing levels over time [33-39]. Therefore, in the case of hereditary breast cancer where protective measures are possible, it may not be necessary to adhere to such a strict counseling protocol as defined for an untreatable neurodegenerative disorder such as HD [32]. Patients with breast cancer express the most interest in answers regarding their personal situation: Is my breast cancer of hereditary origin and what are my children’s risks [submitted data by S. Salemink]? Answers to these questions cannot be given until the results of DNA-testing are known. During the initial face-to-face consultation, patients are provided with general information regarding hereditary breast cancer, DNA-testing and possible consequences, prior to actual DNA-testing. Patients consider this generic information less relevant than the personal advice in the second consultation post DNA-testing and may thus experience this intake as an unnecessary delay [40]. It is also widely known that about 40 to 80% of verbal information is immediately forgotten by patients [41]. A recent study in Canada offered a group of Jewish women DNA-testing through written and telephone invitation. The majority of these women had positive experiences with this approach and considered it to be effective [42]. Providing patients with alternative means to educate themselves regarding hereditary breast cancer and DNA-testing, prior to their decision to undergo testing, might improve patient recollection of medical information as well as increase patient participation.

Therefore, this study offers patients with breast cancer the choice of replacing the initial face-to-face consultation prior to DNA-testing (usual care DNA-intake procedure) by a home information package including telephone, written and digital information consisting of a website and educational movie (DNA-direct procedure). DNA-testing will thus be performed prior to genetic counseling, contrary to current practice. At the first face-to-face contact, counselors will be able to disclose DNA-results and customized advice to patients. This eliminates extraneous information which is not applicable to the individual patient, and provides patients with the information they desire in a quick and patient-centric manner.

The aim of this intervention study is to compare this new DNA-direct procedure to current practice (DNA-intake procedure). The effects of the DNA-direct procedure on the experience and psychosocial distress on patients with breast cancer, as well as the speed and quality of genetic advice, will be evaluated. The hypothesis is that undergoing the DNA-direct procedure does not lead to increased levels of psychosocial distress as compared to the usual care DNA-intake procedure, with equal levels of patient satisfaction plus shorter waiting and processing times. A trend similar to traditional *BRCA1/2* testing – a short term increase in distress, falling back to pre-testing levels over time [33-39] – is expected in the DNA-direct procedure. This would make DNA-direct an acceptable procedure for patients with breast cancer undergoing genetic testing, with the goal of more customized care, and a shorter period of uncertainty. Moreover, it would facilitate taking genetic advice into account for the treatment and follow-up of breast cancer.

## Methods/design

### Design

The study examines the effect of the DNA-direct procedure on the experience and psychological distress of patients with breast cancer as well as several secondary outcome measures, including waiting and processing times, as compared to the current DNA-intake procedure. Two groups will be compared: the intervention group, who choose to undergo the DNA-direct procedure, and the control group, who will receive care as usual (DNA-intake procedure). Participants may choose freely between the DNA-direct versus DNA-intake procedures. This study is not randomized due to the wish to evaluate whether there is indeed a desire for the proposed DNA-direct procedure amongst patients with breast cancer, and to evaluate the reasons stated for preferring one procedure over the other.

### Ethical consideration

The study has been approved by the medical ethical committee of the Radboud University Nijmegen Medical Centre. Full medical ethical approval has been obtained in July 2011.

### Study sample

All female patients previously or currently diagnosed with breast cancer and referred to the department of Clinical Genetics of the Radboud University Nijmegen Medical Centre from August 9th 2011 are eligible for inclusion. Recruitment will continue until the desired total of 150 participants is reached. Patients who have problems reading Dutch text, problems with family communication or problems of psychological/psychiatric nature (including current use of related medication) will be excluded.

### Recruitment

Patients are sent a written letter by a trained doctor announcing a phone call, in which the two choices of procedure are explained (DNA-intake for a face-to-face intake consultation prior to DNA-testing, versus DNA-direct for a home package of telephone, written and digital information) and exclusion criteria are checked. The aim of this telephone approach is triage: by checking for exclusion criteria such as psychological problems, patients who aren’t deemed suitable for DNA-direct (due to its dependency on the patient’s own decision making ability) are filtered out and instead invited for a regular intake consultation, where further psychosocial support is immediately available prior to DNA-testing. Genetic counseling is not offered by phone: questions of this nature are deferred to the personal consultations. The triage phone call has been thoroughly practiced (over 20 times) by the involved doctor with people both specialized and not specialized in clinical genetics.

All patients receive the same two questionnaires, one at inclusion (baseline) and one after completion of the chosen genetic counseling procedure (follow-up). Patients who are confirmed carriers of a *BRCA1/2* mutation, patients reporting problems with the chosen procedure, and randomly selected (n = 10) patients will be invited for a 30 min semi-structured telephone interview.

### *BRCA1* and *BRCA2* genetic testing

The coding sequences and intron/exon boundaries of BRCA1 and BRCA2 are analyzed by sequence analysis (primer sequences available on request). Gross deletions and duplications in the BRCA1 gene are detected by multiplex ligation-dependent probe amplification (MRC-Holland, Amsterdam, kit P002-C2). All findings are confirmed by an independent test.

### Intervention

Patients who choose the DNA-direct procedure, receive a home informational package including an informational letter, a link to a website including a short educational movie about hereditary breast cancer and DNA-testing. Also included are two EDTA blood vials with informed consent and family history forms. Patients are instructed to call their family doctor assistant to ask where to have their blood drawn, then return the vials plus signed forms in the appropriate return package. An appointment for a personal consultation to disclose results is set 8 weeks after DNA-testing has commenced.

All patients (whether they choose DNA-direct or DNA-intake) are seen by one of five selected genetic counselors, each of whom has extensive experience in genetic counseling for hereditary cancer. These counselors have had multiple meetings in order to structure the DNA-direct consultations as follows: If no mutation is found, further screening advice is formulated based on familial risk scores: FHAT [43], Myriad [44] and Claus/van Asperen [45,46]. Further evaluation of family history and features of other hereditary cancer syndromes may be required. In the case of a pathogenic *BRCA1* or *BRCA2* mutation, this result is disclosed immediately, first allowing the patient to react, followed by an explanation of the consequences, including prevention measures and family evaluation. If considered necessary, a second consultation is offered for further genetic counseling. All confirmed *BRCA1/2* carriers will be approached by a social worker to extend psychosocial support if needed, as in usual care.

### Study outcomes

Participants are asked to fill out a questionnaire twice: at baseline and following the conclusion of genetic counseling and/or testing (follow-up). Some measures are used in both questionnaires, while others are only included in either baseline or follow-up.

### Primary outcomes

#### Choice of procedure

Percentages of patients choosing one procedure over the other (ratio between the two groups) is determined to assess the desirability of the new DNA-direct procedure.

#### Psychological burden (baseline and follow-up)

##### Quality of Life

To measure global health-related quality of life (QoL), two items scored on a scale of 1-7 were selected from the EORTC-Q30. The full EORTC-Q30 has been widely used and validated for cancer research [47]. The two global QoL items have an excellent internal consistency as proven by the reported Cronbach’s α of 0.91 [48].

##### General health

The 12-item version of the General Health Questionnaire (GHQ-12) is used as a measure of general psychological distress, using GHQ-scoring of 0,0,1,1 per item (range 0-12) with a threshold of ≥4 to identify ‘caseness', recommended for patients with breast cancer. The GHQ-12 is the shortest version of all GHQs and recommended for research use, with good internal consistency (Cronbach’s α = 0.82 - 0.86) [49-51].

##### Cancer specific distress

The Impact of Event Scale (IES) measures cancer specific distress [52,53] and is included in baseline once using genetic predisposition for cancer as the distressing event, once using breast cancer. For follow-up, only genetic predisposition is included. The IES consists of 15 items, each scored 0,1,3,5. A total score of 9-25 or ≥26 reflects moderate or serious adaptation difficulties respectively. The Dutch version of the IES has a good internal consistency with a Cronbach’s α of 0.87 to 0.96 [53].

##### Risk perception

Risk perception of a genetic predisposition for breast cancer, as well as breast cancer recurrence, is measured on a scale of 0-100.

##### Cancer worry scale

To measure fear of cancer recurrence, the 8-item Cancer Worry Scale (CWS) is included, which has previously been used in studies among cancer patients. Each item is scored 1,2,3,4 from 1 ‘almost never’ to 4 ‘almost always’, the total score ranging from 8-32. It has a good internal consistency with a Cronbach’s α of 0.80 [54-56].

#### Experiences with genetic counseling (follow-up)

##### Decisional conflict

The difficulty of decision-making, in this study defined as whether or not to undergo genetic testing, is assessed using the traditional format of the Decisional Conflict Scale; 1 item (“I expect to stick to my decision”) is left out as it is not applicable to DNA-testing [57-59]. 15 items scored 0,1,2,3,4 from 0 ‘strongly agree’ to 4 ‘strongly disagree’ remained, including “I am satisfied with my decision” which is also used separately for overall satisfaction. Scores are summed, divided by 15 and multiplied by 25, resulting in a range from 0 to 100. Scores below 25 are considered as ‘no decisional conflict’, between 25 and 37.5 as ‘moderate conflict’ and exceeding 37.5 as ‘severe conflict’. The DCS has good internal consistency exceeding 0.78 [57-59].

##### Satisfaction with choice

Knowledge of hereditary breast cancer following versus prior to the chosen genetic counseling procedure, as well as the amount and quality of information received, is rated on a scale of 1-6. Participants answer ‘yes’, ‘no’ or ‘don’t know’ for choosing DNA-testing and/or DNA-direct if given a second chance or asked to give advice to other women in a similar situation.

##### Satisfaction with genetic counseling

The Dutch National Institute for Public Health and the Environment (RIVM) developed a standard questionnaire to measure satisfaction with the service (18 items) and information (8 items) expected of a genetic counselor. Each item is scored on a Likert-scale 1-4, total scores range from 26-104. Open-ended questions evaluate positive/negative experiences during the chosen genetic counseling procedure.

### Secondary outcomes

#### General information (baseline)

##### Demographical and breast cancer information

Data are gathered on age, education level, work status, marital and parental status, cancer status, medical information need (scale 1-10), use of breast cancer information resources and type of information previously given by their referring physician.

##### Empowerment (baseline)

Empowerment is the process in which patients discover and utilize their own power, which will be measured using the Cancer Empowerment Questionnaire (CEQ). It consists of 40 items, each scored on a Likert-scale of 1-5 (1 ‘strongly disagree’, 5 ‘strongly agree’). Four factors are identified: ‘Personal Strength’, ‘Social Support’, ‘Community’ and ‘Health Care’. A good internal consistency (Cronbach’s α = 0.94) was demonstrated for all four factors and the total Empowerment scale [60].

#### Experiences with genetic counseling and testing (follow-up)

##### Choice of procedure

Participants describe reasons for choosing DNA-direct or DNA-intake.

##### Family relations

Three categories of family members are defined: 1) nuclear family, being partner and/or children; 2) family of origin, being parents, brothers and/or sisters; and 3) aunts and female cousins on the family side where breast cancer is prevalent, being the second generation relatives most likely to be affected by genetic testing of the patient. Participants report the frequency of contact with each category of relatives, as well as indicate the quality of their relationship on a scale of 1-10.

##### Family communication

For each above-mentioned relative, participants indicate whether, and if so, when (directly after information, just before DNA-result, after DNA-result) and how often (on a scale of 1-5), they had spoken to this relative about hereditary breast cancer. Also included is the Openness to Discuss Hereditary Cancer in the Family scale (ODHCF) which consists of 7 items each scored 1,2,3,4 with a range 7-24: once for the nuclear family (α = 0.79) and once for the family of origin (α = 0.93) [61].

#### Other measures

Waiting and processing times, as well as family pedigree characteristics, are also gathered.

### Sample size calculation

For this intervention study, participants are not randomized into groups, but given their own choice. This leads to certain complications when it comes to a formal power calculation. First, the ratio between the two procedures is unknown: this may either be balanced (50% versus 50%) or unbalanced (e.g. 20% versus 80%). Second, due to not randomizing, the results will have to be corrected for multiple confounders, which are not all known at this point. The sample size, based on aforementioned ratio between both groups (choice of procedure), needs to be large enough to be able to integrate these confounders into a regression model. Using a power of 80% and a two-tailed probability level for statistical significance testing of 0.05, while taking into account group ratios ranging from balanced (50% versus 50%) versus unbalanced (to an estimated maximum of 20% versus 80%), the total sample size has been set to 150 patients with breast cancer.

### Statistical analysis

To compare general characteristics, baseline and follow-up results between the intervention versus control group, the unpaired *t*-test will be used for continuous variables, Mann–Whitney *U* test for non-parametric variables and chi-square test for dichotomous variables. For the comparison of baseline versus follow-up results within each group, the paired *t*-test will be used for continuous, Wilcoxon test for non-parametric and McNemar’s test for dichotomous variables. Multivariate analysis will consist of a regression model using the follow-up results as outcome (dependent) variables, to be compared between the intervention versus control groups as independent variables, with the baseline results as covariates supplemented by variables that were found to be statistically significant in previous univariate analyses. The probability level for statistical significance testing is set at 0.05 (two-tailed). The SPSS 18.0 statistical package will be used to analyze the data.

## Discussion

Considering today’s call for more patient participation in medical decision-making, the convenience of taking up information and drawing blood close to home, paired with customized advice from the very first consultation, might appeal to patients. Replacing the face-to-face intake consultation with a genetic counselor by a home informational package of telephone, written and digital information might speed up the diagnostic process of hereditary breast cancer and reduce extraneous information. For example, this would allow patients to go over this information at their own convenience and in their own homes. It could possibly reduce travel efforts to a hospital as well as time conflicts with breast cancer therapy.

However, there are certain downsides compared to traditional genetic testing. All patients with breast cancer referred to clinical genetics by their treating physician are eligible for the DNA-direct procedure. This means that even those patients who would not normally fulfill criteria for *BRCA1/2* testing are now able to have their blood drawn for DNA-testing, regardless of those criteria. In the DNA-intake procedure, patients who do not fulfill the aforementioned criteria will not be offered further DNA-testing. This may lead to a selection bias. Our intention is to compare DNA-direct to current practice: adhering to these criteria is the current practice and must be reflected in the DNA-intake procedure.

Additionally, genetic counselors must adjust their counseling styles to the disclosure of the DNA-results being the first order of business, without having built up a counselor-patient relationship beforehand. New information might come forward following result disclosure, leading to ad-hoc modification of screening advice. For this reason, as well as to avoid intercounselor variation, we have selected five genetic counselors with many years of experience in oncogenetic counseling to see all patients participating in this study (both DNA-direct and DNA-intake).

In conclusion, the aim of our study is to determine whether DNA-direct is an acceptable procedure for *BRCA1/2* testing, in order to provide customized care to patients with breast cancer and remove unnecessary waiting times within the diagnostic process, cutting down on the long period of uncertainty that patients are currently faced with.

## Competing interests

The authors declare that they have no competing interests.

## Authors’ contributions

NH and JBP have contributed to the study protocol and revised the manuscript. ASS has contributed to the study protocol and wrote the manuscript. LS, WAGZS, ARM, MJL and HGB have contributed to the study protocol. All authors have read and approved the final manuscript.

## Pre-publication history

The pre-publication history for this paper can be accessed here:

http://www.biomedcentral.com/1472-6874/12/12/prepub
